# Use of ProMED as a Surveillance System for Emerging and Re-Emerging Infectious Diseases in Brazil from 2015 to 2020

**DOI:** 10.3390/v17010093

**Published:** 2025-01-13

**Authors:** Davi Carreiro Rocha, Luana Santos Louro, Hosana Ewald Oliveira, Bruno Cancian de Araujo, Sukhyun Ryu, Creuza Rachel Vicente

**Affiliations:** 1 School of Medicine, Federal University of Espírito Santo, Vitória 29047-105, Espírito Santo State, Brazil; 2 Post-Graduate Program in Infectious Diseases, Federal University of Espírito Santo, Vitória 29047-105, Espírito Santo State, Brazil; 3 Post-Graduate Program in Biotechnology, Federal University of Espírito Santo, Vitória 29047-105, Espírito Santo State, Brazil; brunocanciandearaujo@gmail.com; 4 Department of Preventive Medicine, College of Medicine, The Catholic University of Korea, Seoul 06591, Republic of Korea; gentryu@onehealth.or.kr

**Keywords:** Brazil, emerging or re-emerging infectious diseases, ProMED, surveillance

## Abstract

Emerging and re-emerging infectious diseases have been frequently reported in Brazil. The Program for Monitoring Emerging Diseases (ProMED) is a virtual system with expert curation for monitoring health events, including those occurring in Brazil. This study aimed to describe the ProMED as a complementary surveillance system for emerging infectious diseases in Brazil. It has a retrospective and descriptive design, and was conducted using ProMED-PORT reports that cited Brazil and were published from 1 January 2015, to 31 December 2020. In total, 220 new reports were identified during the study period. Most of these were published between January and June. Reports on humans were predominant (*n* = 177), and comprised 78 kinds of events, most of which were related to arboviruses. Reports on animals were the second most prevalent (*n* = 35), and encompassed 18 kinds of events, particularly yellow fever in non-human primates, rabies in different mammals, and sporotrichosis in felines. Six (2.7%) reports were related to humans and animals, while two (0.9%) were related to plants or the environment. Most reports were from Southeast and Northeast regions. ProMED identified leading emerging and reemerging infectious diseases in Brazil, serving as an information source for local and international health authorities.

## 1. Introduction

In the past decade, Brazil has been frequently affected by emerging and re-emerging infectious diseases. Some of these have become relevant public health concerns, such as Zika, Chikungunya, and coronavirus disease 2019 (COVID-19) [1,2].

Since 1994, the Program for Monitoring Emerging Diseases (ProMED) has supported the early detection of health events. ProMED is an internet-based system with expert curation for the surveillance of emerging infectious diseases, based on different formal and informal information sources [3]. This program aims to complement and not replace other surveillance systems. It focuses on the notification of outbreaks, thereby warning all those interested in preventing and controlling emerging infections in a timely and accurate manner [4,5]. In addition to reports of human diseases, ProMED covers animal and plant diseases [6]. Thus, it applies the One Health approach, which recognizes the interdependence of human, animal, environmental, and plant health, as changes in the ecosystems affect the interactions between pathogens, vectors, and hosts, resulting in the emergence and re-emergence of infectious diseases and the aggravation of existing ones [7,8]. Unfortunately, despite the valuable contribution of ProMED to society, only posts from the previous 30 days have been accessible since July 2023, because of a lack of sustainable funding [9].

Considering the relevance of emerging and re-emerging diseases in Brazil and the importance of comprehensive surveillance systems for their detection, this study aimed to describe ProMED as a complementary surveillance system for emerging infectious diseases in Brazil from 2015 to 2020.

## 2. Materials and Methods

This retrospective and descriptive study was conducted using data from ProMED-PORT reports that cited Brazil and were published from 1 January 2015 to 31 December 2020. ProMED-PORT presents the reports in Portuguese, the official language of Brazil.

The data were accessed through the website www.promedmail.org/promed-posts/ (accessed from 1 August 2021 to 31 December 2021) by using the keyword “Brasil” and the filter ProMED-PORT. These data included reports of diseases identified in Brazil during the study period. The exclusion criteria comprised all reports unrelated to disease events (new reports without events, technical reports, protocol changes, compiled data, comments on articles and research, and other comments), duplicates, reports of event occurrences from a period not included in the study, and reports with insufficient information on the event.

The reports were verified and individually categorized according to the following variables: date of publication, disease, affected individual (human, animal, or plant), state of occurrence, characteristic of the event (epidemic, outbreak, or fatal case), event status (confirmed or suspected), affected population (general or restricted to institutions), and origin of cases (autochthonous or imported).

Simple and relative frequencies were calculated using Microsoft^®^ Excel version 2019.

## 3. Results

Out of the 1754 reports assessed, 136 were excluded: 29 news reports without events, 16 technical reports, 6 protocol changes, 10 compiled data, 25 comments on articles and research, 6 other comments, 18 repeated data, 9 reports of event occurrences from a period not included in the study, and 17 reports with insufficient information on the event. Of the 1618 reports included in this study, 220 (13.6%) were new reports, and 1398 (86.4%) were updates.

**Figure 1:**
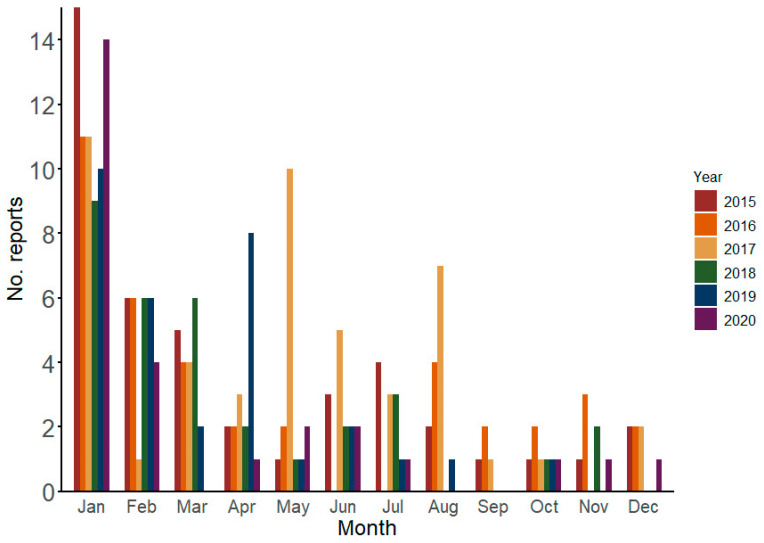
Monthly reports by year in ProMED related to Brazil from 2015 to 2020. Jan = January, Feb = February, Mar = March, Apr = April, Jun = June, Jul = July, Aug = August, Sep = September, Oct = October, Nov = November, Dec = December. Source: ProMED.

The annual average number of new reports was 35 (standard deviation = 7.8), ranging from 27 (2020) to 48 (2017). Most reports (76.8%) were published in the first semester of the year, particularly in January (*n* = 70), February (*n* = 29), March (*n* = 21), April (*n* = 18), and May (*n* = 17) (Figure 1).

Reports of diseases affecting only humans (*n* = 177, 80.5%) or animals (*n* = 35, 15.9%) were predominant. In addition, six (2.7%) reports were related to both humans and animals, while two (0.9%) were related to plants.

**Figure 2:**
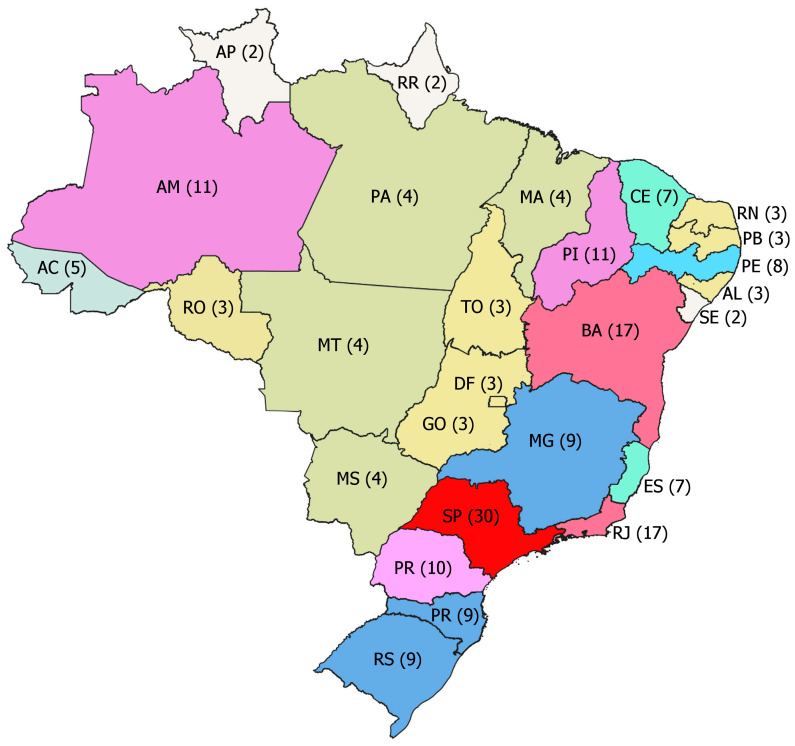
States predominantly cited in reports in ProMED, Brazil, from 2015 to 2020. AC = Acre, AL = Alagoas, AP = Amapá, AM = Amazonas, BA = Bahia, CE = Ceará, DF = Distrito Federal, ES = Espírito Santo, GO = Goiás, MA = Maranhão, MS = Mato Grosso do Sul, MT = Mato Grosso, MG = Minas Gerais, PA = Pará, PB = Paraíba, PR = Paraná, PE = Pernambuco, PI = Piauí, RJ = Rio de Janeiro, RN = Rio Grande do Norte, RS = Rio Grande do Sul, RO = Rondônia, RR = Roraima, SC = Santa Catarina, SP = São Paulo, SE = Sergipe, TO = Tocantins. () = Number of the reports from 2015 to 2020. Data source: ProMED.

The geographical distribution of the reported sites was mainly São Paulo (*n* = 43), Rio de Janeiro (*n* = 18), Bahia (*n* = 17), Amazonas (*n* = 11), Piauí (*n* = 11), Minas Gerais (*n* = 10), Paraná (*n* = 10), Rio Grande do Sul (*n* = 9), Santa Catarina (*n* = 9), Pernambuco (*n* = 8), Espírito Santo (*n* = 6), Ceará (*n* = 6), and Acre (*n* = 5) (Figure 2).

Of the 183 reports on humans, 101 (55.2%) were related to outbreaks, 8 (2.7%) were related to epidemics, and 69 (37.7%) were related to deaths (44 confirmed and 25 suspected). Five events were characterized by an increasing number of disease reports. Moreover, the reports included eight imported cases and seven exported cases. Schools (*n* = 15), prisons (*n* = 6), and hospitals (*n* = 6) were among the most cited institutions in the reports. Indigenous people, military personnel, sheltered people, ship crew, and family members were also mentioned, in two reports each. Measles was the most frequent imported disease, with reports related to Venezuela (*n* = 3) and the United States (*n* = 1). In addition, there was one register of imported Chikungunya from Venezuela, one suspected case of Ebola from Guinea, one case of influenza from France, and one COVID-19 case from China. The exported cases encompassed viral infections, such as yellow fever (one case in 2015 and two cases in 2018), Zika (two in 2016), influenza (one in 2016), and microcephaly (one in 2016).

**Figure 3:**
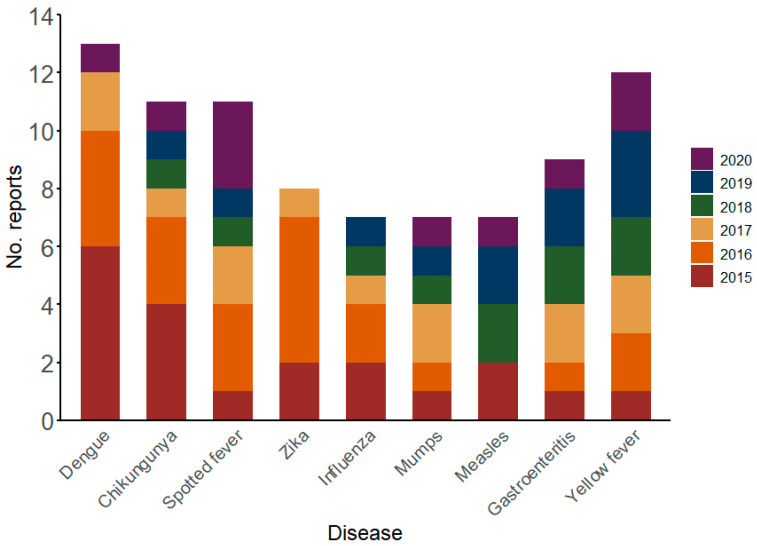
Most reported events in humans, according to ProMED, in Brazil, from 2015 to 2020. Data source: ProMED.

Seventy-eight types of events were reported in humans. The most common events were dengue and Chikungunya in 2015 and 2016; spotted fever in 2016 and 2020; gastroenteritis in 2017, 2018, and 2019; influenza in 2015 and 2016; Zika in 2016; yellow fever in 2019; mumps in 2017; and measles in 2015 and 2019 (Figure 3).

ProMED also identified other emerging conditions, including two reports of microcephaly in 2016 during the Zika outbreak. Reports of Mayaro (2016 and 2019; *n* = 2), West Nile fever (2017–2020; *n* = 4), Oropouche (2017; *n* = 2), *Lomentospora prolificans* infection (2018; *n* = 1), a leishmaniasis-like disease caused by a new parasite (2019; *n* = 1), and COVID-19 (2020; *n* = 1) were also identified.

**Figure 4:**
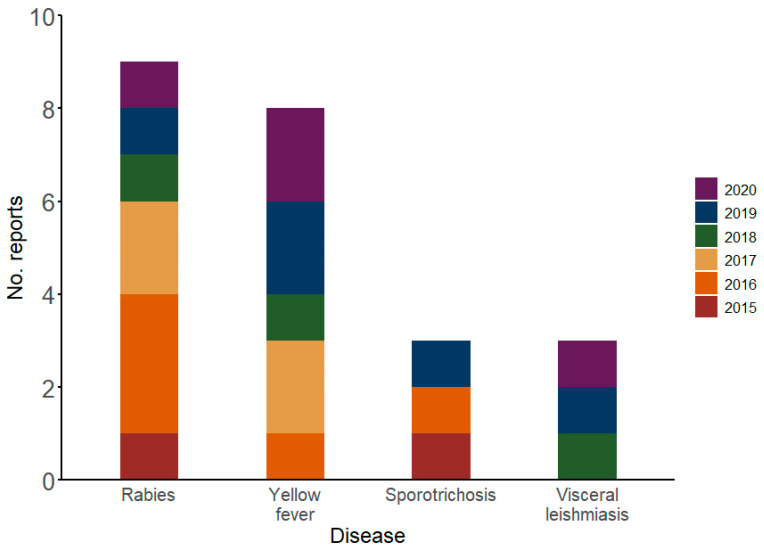
Most reported events in animals, according to ProMED, in Brazil, from 2015 to 2020. Data source: ProMED.

The 41 reports on animals were related to 18 different kinds of events. Primates (*n* = 9; 22%) were the most cited animals and presented with yellow fever (*n* = 8) and herpes (*n* = 1). Cattle (*n* = 7; 17.1%) had rabies (*n* = 4), botulism (*n* = 1), brucellosis (*n* = 1), and bovine spongiform encephalopathy (*n* = 1). Dogs (*n* = 7; 17.1%) presented with visceral leishmaniasis (*n* = 3), distemper (*n* = 2), rabies (*n* = 1), and adverse reaction to rabies vaccine (*n* = 1), while felines (*n* = 4; 9.8%) presented with sporotrichosis (*n* = 3) and rabies (*n* = 1). All three reports on bats were related to rabies (7.3%). Equines (*n* = 3; 7.3%) presented with infectious anemia (*n* = 1), West Nile fever (*n* = 1), and glanders (*n* = 1). All reports on pigs (*n* = 2; 4.9%) were related to swine plague. Other conditions included morbillivirus infection in porpoises (*n* = 1), salmonellosis in fishes (*n* = 1), and tuberculosis in deer (*n* = 1). Rabies was mainly reported in 2016 and 2017; yellow fever in 2017, 2019, and 2020; sporotrichosis in 2015, 2016, and 2019; and visceral leishmaniasis in 2019 and 2020 (Figure 4). Lastly, the two reports on plants were related to coffee berry borer infection (*n* = 1) and citrus canker (*n* = 1).

## 4. Discussion

ProMED identified leading emerging infectious diseases in humans and animals in Brazil, highlighting significant outbreaks and epidemics posing public health concerns locally, nationally, and internationally from 2015 to 2020. Examples include Zika-related microcephaly, declared a public health emergency of international concern in February 2016, and COVID-19, declared a pandemic in 2020 [10,11]. Moreover, reports on yellow fever outbreaks in monkeys and humans were identified after 2016, corroborating the epidemiological alerts from the Pan American Health Organization [12]. A leishmaniasis-like disease caused by a new parasite and the first case of infection by the fungus *L. prolificans* in Brazil were also identified using ProMED.

Diseases in which the etiological agent is transmitted through arthropods, e.g., dengue, Chikungunya, spotted fever, Zika, yellow fever, and visceral leishmaniasis, were most frequently reported in humans and/or animals. Relevant emerging arboviral diseases, such as West Nile fever, Oropouche, and Mayaro, were also reported.

Vector-borne diseases are climate-sensitive [13]. This partially explains the predominance of the reports in the initial months of the year, especially during summer, when higher temperatures and humidity facilitate mosquito and sandfly reproduction [13]. Moreover, most vector-borne diseases are associated with outbreaks and epidemics, explaining the high number of reports on both events [14].

Arboviruses transmitted by the mosquito *Aedes aegypti* were cited in reports throughout the study period, regardless of whether they were previously circulating, such as viruses causing dengue and Chikungunya, or recently introduced, such as Zika virus. Dengue and Chikungunya have been associated with frequent and concomitant epidemics in Brazil, with the country exhibiting the highest absolute incidence of these diseases worldwide [15]. Unsurprisingly, according to EuroTravNet surveillance data (www.eurotravnet.eu), reports of imported cases of these arboviruses in Europe, including those from Brazil, have been increasing in the last 20 years [16]. Moreover, reports of exported cases of Zika and microcephaly were detected in ProMED. It was also possible to identify the end of the Zika outbreak, with no reports detected in ProMED after 2017. The reports on exported arboviruses highlight concerns about their global spread and their local transmission in areas like Southern Europe, where vector populations occur [16]. Climate change also exacerbates this situation, reinforcing the necessity for comprehensive surveillance tools with global epidemiological information, even for non-endemic regions [17].

ProMED also reported that yellow fever was exported to other countries and followed up the epidemiological situation in Brazil, covering epizootics in monkeys and human cases. This event did not involve *A. aegypti* mosquitoes. Instead, transmission occurred in the sylvatic cycle and involved *Haemagogus* mosquitoes [18]. Nevertheless, there is a risk of establishing the transmission in urban areas infested with *A. aegypti* in Brazil and other countries [18]. Consequently, vaccination recommendations have been revised to include all Brazilian territories, including areas without known transmission risk [19]. Thus, ProMED is an updated source for travel-medicine advice.

Spotted fever, an emerging tick-borne rickettsial disease, was also highly reported in humans throughout the study period [20]. Spotted fever partially contributed to São Paulo and Rio de Janeiro being the states with the highest reports in ProMED, with five and three reports of the disease, respectively. Thus, ProMED captured the main transmission areas of spotted fever in Brazil, particularly revealing high incidence and mortality clusters in São Paulo [20].

Gastroenteritis was reported throughout the study period, including outbreaks in schools and prisons and occurrences in different states in Brazil. As gastroenteritis predominantly occurs through contaminated water or food consumption, gastroenteritis outbreaks require immediate investigation from epidemiological surveillance services to interrupt the transmission chain [21]. Thus, ProMED helps disseminate information on these events, in addition to tracking the investigation.

Influenza was also reported frequently, including one report of its exportation to other countries and one of importation. Notably, no report of influenza was detected in ProMED in 2020, suggesting an impact of the COVID-19 pandemic on its notification. The same was observed in the official surveillance system, with few cases of influenza being recorded, indicating a need to improve its molecular surveillance [22]. As ProMED relies on both official and non-official data, a disruption in health systems and a change in news focus, as noted during the COVID-19 pandemic, may affect it considerably.

Vaccine-preventable infections, such as mumps and measles, were among the most reported diseases in ProMED, highlighting the insufficient vaccination coverage in the population, including indigenous people affected by measles and pertussis. Moreover, ProMED captured the re-emergence of measles in Brazil. In particular, there were no reports of measles in 2016 and 2017, when the country was the first in the world to receive the Certificate of Measles Elimination [23]. However, there were new reports in 2018 because of imported cases, especially from Venezuela, in a scenario of reduced vaccination coverage. Brazil thus lost the elimination status in 2019 [23].

Vaccine-preventable diseases were reported in various institutions. These included reports of measles in schools and military units, mumps in prisons and schools, and chickenpox in schools. Thus, ProMED highlighted the necessity of improving vaccination coverage in Brazilian areas to avoid outbreaks in institutionalized populations.

ProMED also covered hospital events, which are sentinels for antimicrobial resistance surveillance. For instance, reports on Carbapenem-resistant *Klebsiella pneumoniae* (CRKP) were detected [24]. Moreover, other hospital-acquired and opportunistic infections were reported, demonstrating the extent of information coverage by ProMED, including data from healthcare facilities.

Among the events reported in animals, rabies was the most frequent event reported throughout the study. It affected diverse mammals, such as cattle, dogs, felines, and bats. Two human cases were also reported. Rabies can be prevented by vaccination. Vaccination campaigns for dogs and cats are conducted periodically in Brazil, and vaccination is also indicated for cattle [25]. Animal vaccination can prevent human cases; however, post-exposure prophylaxis is also essential [25]. Therefore, ProMED identified areas with active transmission of this zoonotic disease where public health actions are required to prevent new cases. Brucellosis, another vaccine-preventable disease affecting cattle, was also reported.

Vector-borne diseases, such as yellow fever and leishmaniasis, were among the most reported diseases in animals. ProMED has reported monkey deaths caused by yellow fever since 2016, following the re-emergence of this disease in Brazil [18]. These deaths are sentinel events that signal the potential occurrence of human infections, and should guide public health actions for prevention and response [18]. Similarly, visceral leishmaniasis in dogs indicates a risk of human cases and involves transmission by phlebotomine sandflies, being constantly reported since 2018 [26]. Moreover, ProMED reported the first case of West Nile fever in equines in Brazil, highlighting the possibility of its spread because of the high presence of the vector *Culex quinquefasciatus* in the Brazilian territory [27].

Sporotrichosis was among the most frequently reported animal infections, predominantly affecting cats. Moreover, two reports were related to humans. Zoonotic transmission of the fungus Sporothrix has been prevalent in Brazil, and a recent increase in transmission in endemic areas has been observed [28]. Considering Brazil’s role in expanding sporotrichosis to other areas of Latin America, primarily through *Sporothrix brasiliensis*, ProMED also contributed to the regional surveillance of this emerging zoonotic disease [28].

Despite the low number of reports on plants or the environment, ProMED described important plant diseases with potential socioeconomic impacts in Brazil, demonstrating its broad coverage of events considering the One Health approach. Plant health surveillance systems are imperative in the One Health context, to ensure food security and environmental health [29].

In addition to the first notification, the number of follow-up notifications on ProMED emphasizes its credibility for surveillance. However, concerns exist regarding confirming and updating events, which are crucial for informing stakeholders, professionals, and the community. Therefore, sustainable funding to support ProMED is vital to guarantee its valuable service to society.

ProMED identified leading emerging and re-emerging infectious diseases in Brazil, serving as an information source for local and international health authorities and helping travel-medicine experts, veterinarians, and individuals dealing with animal trade.

## Data Availability

Data available on www.promedmail.org/promed-posts/.
